# Aetiological agents of pneumonia among HIV and non-HIV infected children in Ghana: A case-control study

**DOI:** 10.1371/journal.pone.0299222

**Published:** 2024-03-22

**Authors:** Michael Owusu, Eric Adu, Lotenna Elsie Kalu, Eugene Martey, Godfred Acheampong, Anthony Enimil, John Adabie Appiah, Augustina Badu-Peprah, Justice Sylverken, Augustina Angelina Sylverken, Samuel Blay Nguah, Emilie Westeel, Stephane Pouzol, Christian Drosten, Yaw Adu-Sarkodie

**Affiliations:** 1 Kumasi Centre for Collaborative Research in Tropical Medicine, Kumasi, Ghana; 2 Department of Medical Diagnostics, Kwame Nkrumah University of Science and Technology, Kumasi, Ghana; 3 Department of Child Health, Kwame Nkrumah University of Science and Technology, Kumasi, Ghana; 4 Centre for Health System Strengthening, Kumasi, Ghana; 5 Department of Radiology, Komfo Anokye Teaching Hospital, Kumasi, Ghana; 6 Department of Theoretical and Applied Biology, Kwame Nkrumah University of Science and Technology, Kumasi, Ghana; 7 Fondation Mérieux, Lyon, France; 8 Charité University, Berlin, Germany; 9 Department of Clinical Microbiology, Kwame Nkrumah University of Science and Technology, Kumasi, Ghana; Nazarbayev University School of Medicine, PAKISTAN

## Abstract

Pneumonia is the leading cause of death in children, however, the microbial aetiology of pneumonia is not well elucidated in low- and middle-income countries. Our study was aimed at determining the microbial aetiologies of childhood pneumonia and associated risk factors in HIV and non-HIV infected children. We conducted a case-control study that enrolled children with pneumonia as cases and non-pneumonia as controls from July 2017 to May 2020. Induced sputum and blood samples were investigated for microbial organisms using standard microbiological techniques. DNA/RNA was extracted from sputum samples and tested for viral and bacterial agents. Four hundred and four (404) subjects consisting of 231 (57.2%) cases and 173 (42.8%) controls were enrolled. We identified a significant (p = 0.011) proportion of viruses in cases (125; 54.1%, 95%CI: 47.4–60.7) than controls (71; 33.6%, 95%CI: 33.6–48.8) and these were mostly contributed to by Respiratory Syncytial Virus. *Staphylococcus aureus* (16; 4.0%), *Klebsiella* spp. (15, 3.7%) and *Streptococcus pneumoniae* (8, 2.0%) were the main bacterial agents identified in sputum or induced sputum samples. HIV infected children with viral-bacterial co-detection were found to have very severe pneumonia compared to those with only viral or bacterial infection. Indoor cooking (OR = 2.36; 95%CI:1.41–3.96) was found to be associated with pneumonia risk in patients. This study demonstrates the importance of various microbial pathogens, particularly RSV, in contributing to pneumonia in HIV and non-HIV paediatric populations. There is a need to accelerate clinical trials of RSV vaccines in African populations to support improvement of patient care.

## Introduction

Lower respiratory tract infections (LRTIs) refer to infections which affect the airways below the epiglottis and could result in severe disease such as pneumonia. Pneumonia is the leading cause of morbidity and mortality in children and adolescents. Despite significant reductions in child mortality over the past two decades, pneumonia still remains among the top five causes of under-five mortality in the sub-Saharan region [1]. The microbial aetiology of pneumonia is variable throughout different population structure, and this is influenced by pathogen endemicity, seasonal climate, geographic location and patient specific characteristics such as human immunodeficiency virus [2, 3].

Pulmonary infections remain the commonest immunodeficiency virus associated illness with complication in the era of antiretroviral therapy (ART) [4]. A systematic review of hospital admissions in HIV-infected patients reported that community acquired pneumonia and tuberculosis collectively accounted for 57% of in-patient deaths globally [5]. Although tuberculosis is often implicated in HIV associated pneumonia, information on the role of other bacterial and viral pathogens is still limited in many developing countries including Ghana. The risk of mortality in hospitalized patients is believed to increase due to limited diagnosis of microbial aetiologies of pneumonia [6]. Effective treatment and management of patients with pneumonia heavily depends on the knowledge of the microbial aetiologies but clinically differentiating between these aetiologies is difficult as they have overlapping presentations. Apart from tuberculosis, it is unclear whether other viral or bacterial organisms found in patients with pnuemonia exist as commensals or pathogens. It is also not known if the presence of these microbial organisms is influenced by HIV infection.

To help improve on this, there have been numerous efforts made especially in developed countries to ascertain the scope of infectious pathogens occurring in HIV-infected patients as compared to non-HIV patients [7]. However, information on the microbial aetiologies of pneumonia in sub-Saharan African countries is still limited. The expansion of molecular diagnostic tests in Africa has improved the identification of microbial pathogens in pneumonia irrespective of the HIV status. The aim of this study was to determine the microbial agents associated with pneumonia in HIV and non-HIV populations and identify other associated risk factors.

## Materials and methods

### Study area and design

This was a prospective, observational case-control study that was conducted at the Komfo Anokye Teaching Hospital from July 2017 to May 2020. The hospital is the second largest in Ghana with 1200 beds. It also serves as the main referral hospital for the Ashanti, Brong-Ahafo and most of the northern parts of Ghana. Recruitment of study participants were done at the Paediatric Emergency Unit (PEU), Paediatric Intensive Care unit (PICU) and Paediatric HIV Clinic (PHC). The PEU takes care of children presenting with medical emergencies. The annual admissions at PEU is about 10,000 with about 21% being pneumonia (Komfo Anokye Teaching Hospital, unpublished). PICU is an 8-bed unit that takes care of children up to 16 years and admits about 102 patients every year. The PHC takes care of confirmed HIV positive patients from birth up to 20 years of age on outpatient basis. In the year 2018, there were approximately 3,025 registered patients in the PHC. The PHC attends to approximately 100 patients a week (Komfo Anokye Teaching Hospital, unpublished).

### Inclusion criteria for cases

Cases were defined as children 3–12 months presenting with clinical symptoms and signs of pneumonia including fever (>37.5°C) and cough with or without difficulty in breathing as well as at least one of the following signs: high respiratory rate (>50 cpm), chest in-drawing, intercostal recession, stridor, pulmonary crackles, lethargy, oxygen saturation < 90% and radiological evidence of pneumonia if available [8]. Cases were sub-classified as severe pneumonia and very severe pneumonia based on the World Health Organization (WHO) classification. Severe pneumonia were patients presenting with cough and/or difficulty in breathing plus fast breathing and chest in-drawings. Very severe pneumonia was defined as cough and/or difficulty in breathing, with one or more of the following signs: central cyanosis or oxygen saturation < 90% on pulse oximetry, severe respiratory distress (e.g., grunting, stridor, severe chest in-drawing) and danger signs of pneumonia (inability to drink or breast feed, head nodding, lethargy or unconsciousness and convulsions) [9, 10].

### Inclusion criteria for controls

Controls were children with comparable age (+/- 3 months) to the recruited cases without history of chronic underlying disease, pneumonia, or respiratory ailment over the past one month prior to screening. Controls were randomly drawn from the same catchment area of the cases and invited to the healthcare facility for sample collection.

### Exclusion criteria for cases and controls

Cases and controls who refused sampling and those outside the catchment area were excluded from the study. Patients positive for tuberculosis and those not on anti-retroviral therapy were also excluded from the study. Controls with history of febrile or respiratory tract illness up to 1 month prior to recruitment were also excluded.

### Screening/Recruitment process

All recruitment processes were carried out by paediatricians and senior resident paediatricians with the support from nurses at PEU, PICU and PHC. For study cases, on arrival at the units, patients with clinical suspicion of pneumonia were considered for the study. A pre-recruitment screening checklist was used to determine eligibility and those who satisfied the criteria were recruited. The study procedure was explained to the parents/guardians and written informed consent was obtained if they agreed to participate. Chest X-rays were done for subjects who had been treated for 48 hours but showed no signs of improvement. The decision to request for chest-x-ray for patients was solely based on the judgement of the paediatric specialist. Chest X-rays were interpreted by a radiologist as normal or abnormal based on standard reporting format. Patients who satisfied the screening procedure were counselled and tested for Human Immunodeficiency Virus (HIV) as part of their routine care. Those positive were referred to the HIV Clinic for subsequent assessment. For every case that was recruited, a healthy control of comparable age was also selected from the neighbouring community of the case. Neighbours and relatives of the cases assisted with this exercise. Controls were selected within 2–4 weeks of recruiting cases.

## Data collection

Biodata collected from the study participants were socio-demographic variables (e.g., age, sex, occupation and educational level of caregivers), clinical information (e.g., weight, height/length, signs and symptoms of pneumonia, presence of complications, radiological features and outcomes of cases) and risk factors (e.g., method of cooking, marital status, mother’s status of breastfeeding and cigarette exposure). Data were collected from the participants using structured paper-based questionnaire.

### Laboratory methods

#### Sample collection

Sputum or induced sputum was collected from both cases and controls. Sputum induction was performed according to guidelines and procedures described by Hammit *et al* and Grant *et al* [11, 12]. Briefly, 2.5 mg/ml of inhaled salbutamol was administered for 10–15 minutes and later followed by 2ml of inhaled hypertonic saline using nebulizer. A minimum volume of about 1ml was collected from patients. The entire sampling procedure was carried out under aseptic conditions.

Sputum and induced sputum samples were transported via cold chain (4°C) to the Kumasi Centre for Collaborative Research in Tropical Medicine (KCCR). At KCCR laboratories, 1ml of the sputum samples were aliquoted into 1ml of RNA later and stored for viral analysis. The leftover was subjected to microbial culture. Blood culture was performed on patients for whom the doctor suspected sepsis in addition to respiratory illness. About 1–3 ml of blood was directly collected into BACTEC bottles from children weighing below 13 kg and 5–8 ml from those 14 kg and above and transported at room temperature to KCCR. Volumes of blood cultures were marked on the bottles before addition of blood and this was observed until the expected mark was reached.

#### Conventional bacteriological investigations

Standard microbiological techniques were performed on sputum and blood culture samples to isolate and identify bacteria from the clinical specimens collected. Sputum samples were pre-treated with sputasol and inoculated by streaking onto chocolate agar (CA), blood agar (BA) and MacConkey agar using sterile disposable loop (10μl). MacConkey and BA were incubated overnight under aerobic conditions at 35–37°C while CA plates were incubated in candle jar and placed in incubator at 35–37°C overnight. Blood cultures were incubated at 35°C using automated BACTEC 9050 system (BD, USA) for a maximum of 7 days. Samples with a positive signal were further processed by inoculating on MacConkey and blood agar plates for further incubation under aerobic conditions (35–37°C).

Morphological characteristics, lactose fermentation, haemolysis on BA, Gram staining and biochemical reactions were used to describe presumptive identification. All Gram-negative bacteria were biochemically confirmed using analytical profile index (API) 20E or 20NE (Biomerieux, France) following all standard microbiological procedures. All Gram-positive bacteria were identified using colony morphology, haemolysis on BA, optochin sensitivity, catalase, and coagulase tests. Streptococci bacteria were speciated using Streptococcal latex grouping kits (Oxoid, UK). *Escherichia coli* ATCC 25922, *Salmonella* Typhimurium ATCC 14028, *Staphylococcus aureus* ATCC 25923 and *Streptococcus pneumoniae* ATCC 254697 were set up together with the test organisms to control media and biochemical tests.

### Viral testing using realtime—PCR

Nucleic acids were purified from the sputum samples using Qiagen Viral RNA Mini Kit according to the manufacturer’s protocol (Qiagen, Hilden, Germany). Purified nucleic acids were tested for Respiratory Syncytial Virus (RSV), Influenza A and B (INF A, B), Parainfluenza 1, 2, 3 (PIV 1, 2, 3), Adenoviruses, Human Coronaviruses (OC43, HKU1, NL63, 229E), Human metapneumovirus (hMPV) and Rhinoviruses (HRV). SARS-CoV-2 test was performed on RNA extracts in retrospect during the outbreak of COVID-19. The Qiagen One Step RT-PCR kit was used for testing following manufacturer’s protocol. Briefly, a 25μl reaction mixture consisting of optimized volumes of deoxynucleotide triphosphate, magnesium chloride, Qiagen One Step Buffer and enzyme mix was reconstituted for virus detection. Samples were pooled together and singleplex RT-PCR was performed with primers and probes highly specific to the target regions of the viruses. SARS-CoV-2 testing was performed on the samples using DaAnGene Detection Kit for 2019 n-CoV (Guangdong, China). The SARS-CoV-2 PCR kit was designed to target the ORF1ab and nucleocapsid regions of the virus. Test results for each virus was interpreted as positive based on cut-off values of the cycling threshold stated in the manufacturer’s protocol. The PCR cycling conditions for the other viruses were done as described by other authors (Table 1).

**Table 1:** Primers and probes used for the molecular detection of respiratory viruses.

Virus type	Target region	Function	Sequence	Reference
RSV(A/B)	Matrix gene	Forward primer	5’-GGAAACATACGTGAACAAGCTTCA	[13]
		Reverse primer A	5’-CATCGTCTTTTTCTAAGACATTGTATTGA	
		Reverse primer B	5’-TCATCATCTTTTTCTAGAACATTGTACTGA	
		Probe	6FAM-TGTGTATGTGGAGCCTT- MGBNFQ	
Adenovirus	Hexon gene	Forward primer	5’-GCCACGGTGGGGTTTCTAAACTT	[14]
		Reverse primer	5’-GCCCCAGTGGTCTTACATGCACAT	
		Probe	6FAM-GCACCAGACCCGGGCTCAGGTACTCCGA-TAMRA	
INF A	Matrix gene	Forward primer	5’- GACCRATCCTGTCACCTCTGAC	[15]
		Reverse primer	5’-AGGGCATTYTGGACAAAKCGTCTA	
		Probe	5’-TGCAGTCCTCGCTCACTGGGCACG	
INF B	Hemagglutin (HA) gene	Forward primer	5’-AAATACGGTGGATTAAATAAAAGCAA	[16]
		Reverse primer	5’-CCAGCAATAGCTCCGAAGAAA	
		Probe	6FAM-CACCCATATTGGGCAATTTCCTATGGC- MGBNFQ	
HRV	Non-coding region	Forward primer	5’-CPXGCCZGCGTGGC	[17]
		Reverse primer	5’-GAAACACGGCACCCAAAGTA	
		Probe	5’-TCCTCCGGCCCCTGAATGYGGC	
hMPV	Nucleoprotein	Sense	5’-CATCAGGTAATATCCCACAAAATCAG-3’	[18]
		Antisense	5’-GTGAATATTAAGGCACCTACACATAATAARA-3’	[18]
		Probe	6FAM-TCAGCACCAGACACAC-BBQ	
OC43	Nucleoprotein	Forward primer	5’-CGATGAGGCTATTCCGACTAGGT	[19]
		Reverse primer	5’-CCTTCCTGAGGTAACC	
		Probe	5’-TCCGCCTGGCCTCCCT	
229E	Nucleoprotein	Forward primer	5’-CAGTCAAATGGATGCA	[19]
		Reverse primer	5’-AAAGGGCTATTATTCT	
		Probe	5’-CCCTGACGACGGTTCA	
NL63	Nucleoprotein	Forward	GACCAAAGCACTGAATAACATTTTCC	[19]
		Reverse	ACCTAATAAGCCTCTTTCTCAACCC	
		Probe	6FAM-ATGTTATTCAGTGCTTTGGTCCTCGTGAT-BHQ1	
		Probe	5’-TGTGTGGCGGAGCCTG	
HKU1	Replicase gene	Forward	CCTTGCGAATGAATGTGCT	[19]
		Reverse	TTGCATCACCACTGCTAGTACCAC	
		Probe	6FAM-TGTGTGGCGGTTGCTATTATGTTAAGCCTG-BHQ1	
PIV 1	Polymerase gene	Forward primer	5’-ACAGATGAAATTTTCAAGTGCTACTTTAGT	[20]
		Reverse primer	5’-GCCTCTTTTAATGCCATATTATCATTAGA	
		Probe	6FAM-ATGGTAATAAATCGACTCGCT- MGBNFQ	
PIV 2	Polymerase gene	Forward primer	5’-TGCATGTTTTATAACTACTGATCTTGCTAA	[20]
		Reverse primer	5’-GTTCGAGCAAAATGGATTATGGT	
		Probe	6FAM-ACTGTCTTCAATGGAGATAT- MGBNFQ	
PIV 3	Matrix gene	Forward primer	5’-TGCTGTTCGATGCCAACAA	[20]
		Reverse primer	5’-ATTTTATGCTCCTATCTAGTGGAAGACA	
		Probe	6FAM-TTGCTCTTGCTCCTCA- MGBNFQ	

### Ethical consideration

Ethical approval was sought and obtained from the Committee on Human Research Publication and Ethics (CHRPE) of the School of Medicine and Dentistry, Kwame Nkrumah University of Science and Technology, Kumasi (Approval numbers: CHRPE/AP/389/17; CHRPE/AP/537/18; CHRPE/AP/530/19) and permission sought from the Komfo Anokye Teaching Hospital before commencement of the study. Confidentiality was ensured by issuing participants with unique identification numbers, with the patients’ names omitted. Written informed consent were provided by parents or guardians prior to recruitment of their children.

### Sample size consideration

Prior to undertaking this study, we estimated a samples size of 179 individuals each for cases and controls. This estimate was based on previous RSV detections of 21.3% being the most predominant virus among children and estimated 10% exposure among controls (Kwofie et al., 2018) [21]. The estimated sample size determined was based on a power of 80% and a two-sided alpha error of 5%.

### Statistical analysis

Data were entered into Microsoft Excel and exported to R statistical software (version 3.6.1) for analysis. Descriptive statistics were computed for continuous and categorical variables. Statistical comparisons between subgroups of categorical variables were analyzed using the Fischer’s exact test or Chi-square test where appropriate. A multivariate logistic regression was used to determine the association between microorganisms detected and pneumonia risk. Multivariate models were adjusted for microorganisms detected in induced sputum separately for HIV and non-HIV patients. Results were expressed as odds ratio with 95% confidence interval. For all analysis, a p-value of less than 0.05 was considered statistically significant.

## Results

### Characteristics of study participants

The study recruited 404 individuals with males being 232 (58.3%). Of the 404 recruited, 148 (36.6%) were HIV infected persons and among them 57.4% (85/148) presented with pneumonia. A comparison of the socio-demographic variables and other risk variables among non-HIV populations showed history of breastfeeding, indoor cooking, marital status and mode of cooking methods were associated with pneumonia. Among HIV populations we observed, guardians’ highest educational level, exposure to cigarette and mode of cooking were associated with pneumonia. Table 2 gives a breakdown of the socio-demographic variables their association with the pneumonia.

**Table 2:** Socio-demographic characteristics of HIV and non-HIV cases and controls.

	Non-HIV infected children				HIV-infected children			
	Non-Pneumonia Controls	Pneumonia Cases	Total	P value	Non-Pneumonia Controls	Pneumonia Cases	Total	P value
Total	110	146	256		63	85	148	
**Gender**				0.541				0.392
Female	48 (43.6)	57 (39)	105 (41)		29 (46)	32 (37.6)	61 (41.2)	
Male	62 (56.4)	89 (61)	151 (59)		34 (54)	53 (62.4)	87 (58.8)	
**Age categories in months**				0.097				0.673
3–5 months	71 (64.5)	78 (53.4)	149 (58.2)		34 (54)	50 (58.8)	84 (56.8)	
6–12 months	39 (35.5)	68 (46.6)	107 (41.8)		29 (46)	35 (41.2)	64 (43.2)	
**Ever Breastfed**	74 (67.9)	50 (35.7)	124 (49.8)	**< 0.001**	49 (94.2)	67 (91.8)	116 (92.8)	0.734
**Schooling**	6 (5.7)	15 (11.5)	21 (8.9)	0.178	27 (46.6)	51 (63)	78 (56.1)	0.08
**Marital status**				**0.009**				0.83
Married	71 (67)	108 (77.7)	179 (73.1)		22 (48.9)	34 (51.5)	56 (50.5)	
Co-habitation	32 (30.2)	21 (15.1)	53 (21.6)		7 (15.6)	12 (18.2)	19 (17.1)	
Others	3 (2.8)	10 (7.2)	13 (5.3)		16 (35.6)	20 (30.3)	36 (32.4)	
**Cigarette exposure**	16 (15.7)	21 (15.3)	37 (15.5)		6 (10.2)	23 (28)	29 (20.6)	
**Patients’ mode of cooking**				**0.002**				0.937
Indoor Cooking	52 (47.7)	97 (68.3)	149 (59.4)		28 (46.7)	40 (48.8)	68 (47.9)	
Outdoor Cooking	57 (52.3)	45 (31.7)	102 (40.6)		32 (53.3)	42 (51.2)	74 (52.1)	
**Cooking method**				**< 0.001**				**< 0.001**
Charcoal	0 (0)	34 (26.4)	34 (14.3)		0 (0)	23 (28)	23 (16.1)	
Gas Stove	109 (100)	83 (64.3)	192 (80.7)		61 (100)	51 (62.2)	112 (78.3)	
Firewood	0 (0)	12 (9.3)	12 (5)		0 (0)	8 (9.8)	8 (5.6)	

### Microbial pathogens distribution

#### Bacterial organisms in blood

Of the 404 patients, blood cultures were performed on 194 pneumonia cases who presented with signs and symptoms suggestive of sepsis as determined by the attending physician. The prevalence of pathogenic bacteria isolation in blood culture was 5.7% (11/194). The organisms isolated were *Acinetobacter* spp. (1; 0.5%), *E*. *coli* (2; 1.0%), *Pseudomonas* spp. (2; 1.0%), *Salmonella* spp. (3, 1.5%), *Staphylococcus aureus* (2; 1.0%) and Group F streptococci (1; 1.0%). There was no difference between the proportion of bacteria isolated in the blood culture of HIV patients (4, 5.1%) compared to non-HIV (7, 6%).

#### Bacterial organisms in sputum and induced sputum

Of the 404 patients recruited, bacteria organisms were isolated in 62 subjects (15.3%). The commonest bacteria isolated was *Klebsiella* spp. (15, 3.7%), followed by *Staphylococcus aureus* (16; 4.0%) and *Streptococcus pneumoniae* (8, 2.0%). The overall proportion of bacteria in pneumonia cases (46, 20%) was significantly different (p = 0.005) than that of controls (16, 9.2%). There was no significant difference in the overall rate of bacteria identified in the HIV cases (19, 22.4%) compared to non-HIV cases (25, 17.1%). Subgroup analysis however identified *Pseudomonas aeruginosa* as being predominant in HIV cases (5, 5.9%) compared to non-HIV cases (0) but the difference was not significant. On the other hand, *Staphylococcus aureus* was more common in non-HIV cases (9, 6.2%) compared to HIV cases (0) while *Klebsiella* spp. was predominant in cases (14, 6.1%) compared to controls (1, 0.6%). *Klebsiella* spp. was independently associated with pneumonia risk. Of all patients tested, we observed only one patient had *Staphylococcus aureus* isolated in both blood and sputum. Table 3 gives details of pathogens identified among study subjects.

**Table 3:** Bacteria distribution in sputum/induced sputum samples.

	Cases				Overall Cases and Controls			
	Non-HIV	HIV	AOR^a^ (95%CI)	P value	Pneumonia Cases	Non-Pneumonia Controls	AOR^b^ (95%CI)	P value
**Total**	**146**	**85**			**231**	**173**		
***Acinetobacter* spp.**	0 (0)	1 (1.2)	N/A	0.112	1 (0.4)	0 (0)	N/A	0.284
***E*. *coli***	1 (0.7)	1 (1.2)	0.56 (0.03,9.27)	0.689	2 (0.9)	1 (0.6)	1.52 (0.14,17.12)	0.729
***Enterobacter* spp.**	1 (0.7)	1 (1.2)	0.62 (0.04,10.19)	0.739	2 (0.9)	0 (0)	N/A	0.128
***Klebsiella* spp.**	9 (6.2)	5 (5.9)	0.89 (0.28,2.84)	0.844	14 (6.1)	1 (0.6)	10.91 (1.41,84.5)	**0.002**
***Methylobacterium* spp.**	0 (0)	1 (1.2)	N/A	0.996	1 (0.4)	0 (0)	N/A	0.265
***Pseudomonas aeruginosa***	0 (0)	5 (5.9)	N/A	0.992	5 (2.2)	1 (0.6)	4.32 (0.5,37.61)	0.129
***Staphylococcus aureus***	9 (6.2)	0 (0)	N/A	0.99	9 (3.9)	7 (4)	1.11 (0.4,3.08)	0.835
***Streptococcus pneumoniae***	4 (2.7)	3 (3.5)	0.73 (0.16,3.37)	0.684	7 (3)	1 (0.6)	6.72 (0.81,55.88)	0.078
***Streptococcus* spp.**	1 (0.7)	1 (1.2)	0.48 (0.03,7.85)	0.609	2 (0.9)	1 (0.6)	1.43 (0.13,16.18)	0.769

N/A: Could not estimate values due to low numbers.

^a^Odds ratio adjusted for other viral organisms predicting pneumonia in non-HIV patients compared to HIV patients

^b^Odds ratio adjusted for other viral agents predicting for pneumonia cases compared to controls.

#### Viral distribution

We tested for 14 different viruses among 404 patients consisting of 231 pneumonia cases and 173 controls. Of all patients tested, the most common virus identified was HRV (89, 22.1%), followed by RSV (52, 12.9%), Adenovirus (49, 12.2%), Influenza A (24, 6%), HCoV-HKU1 (10, 2.5%), HCoV-NL63 (8, 2%), HCoV-OC43 (5, 1.2%), PIV-1 (2, 0.5%) and Influenza B (5, 1.2%). PIV-3 and SARS-CoV-2 were not detected in the samples. Viral co-infections occurred for Adenovirus and HRV (20, 5%); Adenovirus and Influenza A (2, 0.5%); HRV and RSV (11, 2.7%) and Influenza A and RSV (6, 1.5%). The total number of viruses identified in the cases (125, 54.3%) was higher than controls (71, 41%) and the difference was statistically significant (p<0.01). RSV and Influenza viruses were the most common in cases compared to controls. RSV was found to be independently associated with the odds of developing pneumonia (6.85, 95%CI = 2.81–16.71), especially in non-HIV cases with higher odds (7.49, 95%CI:2.55–21.98) compared to HIV patients. Table 4 gives description of viral pathogens identified.

**Table 4:** Virus distribution in cases and controls.

	Pneumonia Cases				Cases and Controls			
	Non-HIV Patients	HIV Patients	AOR^a^ (95%CI)	P value	Pneumonia Cases	Non-Pneumonia Controls	AOR^b^(95%CI)	P value
**Total**	**146**	**85**			**231**	**173**		
**ADENO**	15 (10.3)	14 (16.7)	0.83 (0.34, 2.07)	0.698	29 (12.6)	20 (11.6)	1.2 (0.63, 2.44)	0.531
**HKU1**	2 (1.4)	4 (4.8)	0.35 (0.06, 2.01)	0.225	6 (2.6)	4 (2.3)	1.3 (0.35, 4.84)	0.703
**HCoV-229E**	3 (2.1)	2 (2.4)	0.77 (0.11, 5.19)	0.787	5 (2.2)	0 (0)	N/A	0.987
**HCoV NL63**	3 (2.1)	2 (2.4)	0.84 (0.12, 5.67)	0.854	5 (2.2)	3 (1.7)	1.3 (0.3, 6.13)	0.70
**HCoV OC43**	0 (0)	2 (2.4)	N/A	0.208	2 (0.9)	3 (1.7)	0.46 (0.07, 3.12)	0.43
**HMPV**	1 (0.7)	0 (0)	N/A	0.31	1 (0.4)	0 (0)	N/A	-0.99
**HRV**	27 (18.5)	18 (21.4)	0.79 (0.37, 1.67)	0.536	45 (19.6)	44 (25.4)	0.69 (0.41, 1.15)	0.157
**INF.A**	13 (8.9)	6 (7.1)	0.99 (0.33, 2.93)	0.982	19 (8.3)	5 (2.9)	2.45 (0.86, 6.99)	0.094
**INF.B**	0 (0)	3 (3.6)	N/A	0.94	3 (1.3)	2 (1.2)	1.62 (0.25, 10.56)	0.613
**RSV**	42 (28.8)	4 (4.8)	7.49 (2.55, 21.98)	**<0.001**	46 (20)	6 (3.5)	6.85 (2.81, 16.71)	**< 0.001**
**PIV.1**	2 (1.4)	0 (0)	N/A		2 (0.9)	4 (2.3)	N/A	0.376
**PIV.2**	1 (0.7)	1 (1.2)	0.93 (0.05, 16.12)	0.96	2 (0.9)	0 (0)	N/A	0.097

^a^Odds ratio adjusted for other viral organisms predicting pneumonia in non-HIV compared to HIV

^b^Odds ratio adjusted for other viral agents predicting for pneumonia cases compared to controls. NA: Could not estimate values due to low numbers.

### Association between chest X-ray findings and microbial detection

As part of this study, we collected chest X-ray information on patients and correlated this with selected viral or bacterial organisms. Of the 231 pneumonia cases enrolled, chest X-rays were done for 149 (64.5%). Of these, 72 (48.3%) were abnormal. The common abnormalities were alveolar (42, 28.2%) and interstitial (22, 15.4.%) infiltrates. Others were mainly pleural diseases including effusion, pneumothorax and hydropneumothorax. For all the pathogens identified, we observed influenza A was significantly associated with abnormal chest x-ray. Further stratification based on HIV status did not show any difference in x-ray abnormality for all pathogens except for Influenza A which was found to be associated with abnormal chest-X-ray in non-HIV patients (Table 5).

**Table 5:** Chest X-rays findings for various pathogens detected.

	Abnormal	Normal	Total	P value
**Total**	**72**	**77**	**149**	** **
*Klebsiella* spp.	4 (5.6)	5 (6.5)	9 (6)	1
*S*. *pneumonia*	3 (4.2)	2 (2.6)	5 (3.4)	0.673
*S*. *aureus*	5 (6.9)	1 (1.3)	6 (4)	0.107
INF.A	9 (12.5)	1 (1.3)	10 (6.7)	**0.008**
RSV	12 (16.7)	17 (22.1)	29 (19.5)	0.531
ADENO	11 (15.3)	10 (13)	21 (14.1)	0.868
HRV	15 (20.8)	16 (20.8)	31 (20.8)	1
229E	0 (0)	5 (6.5)	5 (3.4)	0.059
NL63	1 (1.4)	2 (2.6)	3 (2)	1

### Association between clinical presentations and microbial detections

We explored other clinical variables and outcomes associated with viral detection, bacterial infections and viral-bacterial co-infections. We observed significant association between patients having viral-bacterial co-infections and lower chest-indrawing (p = 0.04) and very severe pneumonia (p = 0.017) (S1 Table). Among HIV cases, we observed significant variations between viral-bacterial co-infections and clinical manifestations including poor feeding, fast breathing, chest recession, lower chest in-drawings and very severe pneumonia. Pulmonary crackles were more associated with viral infections (S2 Table). We did not observe significant association between non-HIV populations and microbial detection (S3 Table). A sub-level analysis of the individual viruses (RSV, Influenza A and others) and bacteria did not also show any significant association with the clinical presentations.

### Seasonality of microbial detections

**Fig 1:**
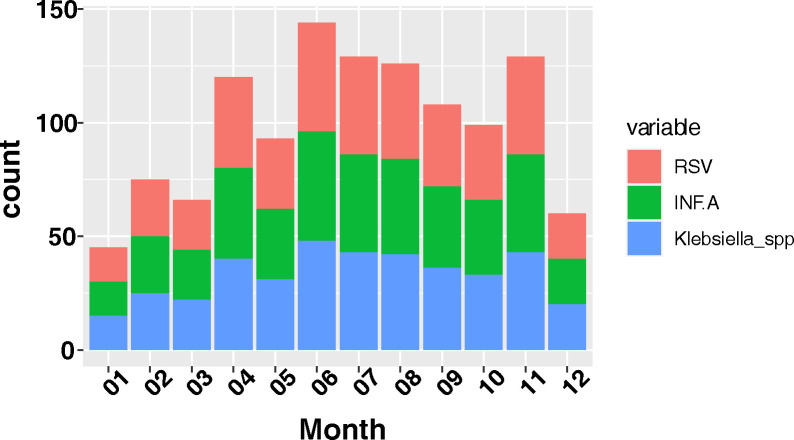
A graph showing the seasonality of microbes detected in the study.

In order to ascertain the seasonal variations of microbial detections over the study period, we plotted the top three microbial organisms (influenza A, RSV and *Klebsiella* spp.) by the date of enrolment of patients. We observed significant variations in RSV detections (p = 0.001) with most infections occurring in the month of June and July. Influenza A viral infections also occurred frequently in June and July however the variations across the various months was not significant. *Klebsiella* spp. was almost evenly distributed over the various months (Fig 1).

### Patient outcomes

Of the 231 pneumonia cases enrolled, 3 (0.74%) patients died at the end of the study. All 3 cases of death had a median age of 4 months (IQR = 3.5–6) and needed oxygen within the first 48 hours of admission. The deaths occurred among non-HIV subjects with pneumonia. One of the 3 patients who died had only *Staphylococcus aureus* isolated in the induced sputum, and another had both *Staphylococcus aureus* and influenza A co-infection.

## Discussion

Of the various infectious agents of pneumonia, viral associated pneumonia is a major public health problem due to their ease of transmission, substantial morbidity, and wide occurrence. Our study identified common viral agents including RSV, Adenoviruses, influenza A and human coronaviruses in the study population. We did not record any case of SARS-CoV-2 because the samples were collected before the outbreak of SARS-CoV-2 in Ghana.

Higher proportion (54.3%) of viruses was observed among patients with pneumonia compared to controls with RSV being most predominant. This prevalence is higher than what was reported from our previous research conducted 10 years ago at the same study site, in which there was virus isolation in 25.8% of pneumonia patients [21]. A possible explanation for this discrepancy could be the utilization of induced sputum samples which some studies have reported to provide better microbial yield than nasopharyngeal samples [22, 23]. We observed control subjects had frequent detections of HRV and Adenoviruses contributing mostly to 41% of controls. This observation has been reported by other authors where HRV and Adenoviruses are deemed to be more common in health individuals and may not be of any clinical value causing disease. The younger age group in this current study could also explain the increased detections of RSV compared to our previous study [21]. The previous study enrolled children less than 5 years of age while this current study enrolled children less than 12 months.

RSV was observed as the main virus associated with pneumonia in both HIV and non-HIV patients. The implications of RSV in pneumonia has been reported by other authors [24, 25]. Our study did not however record significant association between RSV infections and clinical manifestations perhaps due to the low number of case groups enrolled. Other studies have however recorded more severe and prolonged symptoms with increased risk of complications especially in very young children [26–29]. In both developed and developing countries, wheezing has also been reported [30, 31].

The increased detections of RSV in children presenting with pneumonia emphasise the need for interventions to reduce the burden of disease in Africa. Currently there is no specific treatment for RSV. Only supportive care measures like oxygen and fluids exist. Vaccine to prevent RSV is also not widely available to the public. However, there are reports of Phase 3 trials of maternal RSV vaccines which have shown 82% effectiveness in preventing severe lower respiratory tract infections due to RSV in newborns up to 90 days after birth, and 69% effective for the first 6 months after birth, [32] giving hopes of possible implementation globally in the near future.

Assessment of sputum or induced sputum specimens using conventional cultures revealed a high number of bacterial organisms in pneumonia cases compared to controls. The most predominant microbial organism isolated was *Klebsiella* spp., followed by *Streptococcus pneumoniae* and *Staphylococcus aureus*. For both HIV and non-HIV populations, *Klebsiella* spp. was found to be significantly predominant in cases compared to controls. *Klebsiella* spp. is reported as one of the most common bacterial agents of community acquired pneumonia [33]. Klebsiella inclusively was associated with 63.4% of deaths from infectious diseases, including acute and secondary pneumonia, post-traumatic and post-operative complications and sepsis [34]. *Klebsiella* spp. strains were found to be three times more prevalent than pneumococci and more than six times prevalent than other types of bacteria [34]. We also observed other common microbial organisms including *Streptococcus pneumoniae* in pneumonia cases and *Pseudomonas aeruginosa* in HIV patients but these were not significant. Other studies have indicated the important role of these organisms in contributing to severe and very severe pneumonia [35].

Conversely to single viral or bacterial infection, viral-bacterial co-infection was found to be significantly associated with very severe pneumonia. This association was found to be more pronounced in HIV patients compared to non-HIV patients. Determining the contribution of mixed infection to disease severity is highly complex, particularly among the pediatric population. Despite these complexities, many clinical studies on co-infection seem to provide evidence of enhanced disease severity during polymicrobial acute respiratory tract infection in children [36, 37]. Quah et al., recently reported viral-bacterial co-infections were associated with increased risk of hospital mortality (OR = 13.99; 95% CI = 1.3–151.05) [38]. The severity of clinical disease could be explained by the ability of mixed viral-bacterial co-interactions that are known to modulate the JAK-STAT signaling pathway which is believed to exacerbate IP-10 expression [39]. This therefore suggest the importance of managing these microbial organisms when isolated from patients who show clear signs of pneumonia.

In terms of socio-demographic characteristics of our study populations, we observed HIV infected children exposed to cigarette and non-HIV children involved in indoor cooking and other modes were more associated with pneumonia. Indoor air pollution is one of the leading risk factors of childhood pneumonia in developing countries. Exposure to environmental tobacco smoke is a major source of fine and respirable particles in indoor environment. Parental smoking, use of solid fuel and other indoor activities such as use of mosquito coils are reported to increase the risk of pneumonia. This phenomenon has been reported in other developing countries [40, 41].

### Limitations of study

One major limitation of the study is our inability to use molecular techniques to investigate the full spectrum of non-viral microbial organisms. We did not also perform conventional cultures for fungal agents. The use of these advanced methods could have allowed for proper estimation of the full spectrum of other non-viral agents involved in the causal pathway of pneumonia. Nevertheless, the conventional techniques used provides baseline information that could guide future studies. The difficulty in enrolling healthy people to provide induced sputum limited our ability to recruit large number of persons for this study. An imbalance in the case-control ratio numbers could have impact on the results.

## Conclusion

This study has emphasised the importance of microbial organisms particularly RSV in contributing to pneumonia among children with/without HIV. Identification of these pathogens has improved our knowledge on the burden of respiratory pathogens in Africa. The study has provided further evidence about the need for further advocacy for vaccines to improve the wellbeing of children in Africa.

## Supporting information

S1 Checklist STROBE statement—checklist of items that should be included in reports of observational studies. (DOCX)

S1 Table Clinical presentations associated with microbial detection for all cases. (PDF)

S2 Table Clinical presentation associated with microbial detection in HIV patients with pneumonia. (PDF)

S3 Table Clinical presentation associated with microbial detection in non-HIV patients. (PDF)

## References

[1] WHO. Pocket book of Hospital Care for Children: guidelines for the management of common childhood illnesses. In Pneumonia. 2 nd ed. Geneva, Switzerland:2013. 24006557

[2] Bunthi C, Rhodes J, Thamthitiwat S, Higdon MM, Chuananon S, Amorninthapichet T, et al. Etiology and Clinical Characteristics of Severe Pneumonia Among Young Children in Thailand: Pneumonia Etiology Research for Child Health (PERCH) Case–Control Study Findings, 2012–2013. The Pediatric infectious disease journal. 2021;40(9):S91. doi: 10.1097/INF.0000000000002768 34448748 PMC8448397

[3] Bénet T, Sánchez Picot V, Messaoudi M, Chou M, Eap T, Wang J, et al. Microorganisms associated with pneumonia in children< 5 years of age in developing and emerging countries: the GABRIEL pneumonia multicenter, prospective, case-control study. Clinical Infectious Diseases. 2017;65(4):604–12. 28605562 10.1093/cid/cix378 PMC7108107

[4] Benito N, Moreno A, Miro J, Torres A. Pulmonary infections in HIV-infected patients: an update in the 21st century. European Respiratory Journal. 2012;39(3):730–45. doi: 10.1183/09031936.00200210 21885385

[5] Ford N, Shubber Z, Meintjes G, Grinsztejn B, Eholie S, Mills EJ, et al. Causes of hospital admission among people living with HIV worldwide: a systematic review and meta-analysis. The lancet HIV. 2015;2(10):e438–e44. doi: 10.1016/S2352-3018(15)00137-X 26423651

[6] Wasserman S, Engel ME, Griesel R, Mendelson M. Burden of pneumocystis pneumonia in HIV-infected adults in sub-Saharan Africa: a systematic review and meta-analysis. BMC infectious diseases. 2016;16(1):1–9. doi: 10.1186/s12879-016-1809-3 27612639 PMC5018169

[7] Dillon S, Lee E, Kotter C, Austin G, Dong Z, Hecht D, et al. An altered intestinal mucosal microbiome in HIV-1 infection is associated with mucosal and systemic immune activation and endotoxemia. Mucosal immunology. 2014;7(4):983–94. doi: 10.1038/mi.2013.116 24399150 PMC4062575

[8] Gove S. Integrated management of childhood illness by outpatient health workers: technical basis and overview. The WHO Working Group on Guidelines for Integrated Management of the Sick Child. *Bull World Heal Organization*. 1997;75 Suppl 1:7–24. PMC2486995 9529714

[9] Scott JAG, Wonodi C, Moïsi JC, Deloria-Knoll M, DeLuca AN, Karron RA, et al. The definition of pneumonia, the assessment of severity, and clinical standardization in the Pneumonia Etiology Research for Child Health study. Clinical infectious diseases. 2012;54(suppl_2):S109–S16. doi: 10.1093/cid/cir1065 22403224 PMC3297550

[10] WHO. Pocket book of hospital care for children: Second edition: Guidelines for the management of common childhood illnesses2017 November 28, 2017.

[11] Hammitt LL, Murdoch DR, Scott JAG, Driscoll A, Karron RA, Levine OS, et al. Specimen collection for the diagnosis of pediatric pneumonia. Clinical infectious diseases. 2012;54(suppl_2):S132–S9. doi: 10.1093/cid/cir1068 22403227 PMC3693496

[12] Grant LR, Hammitt LL, Murdoch DR, O’Brien KL, Scott JA. Procedures for collection of induced sputum specimens from children. Clinical infectious diseases. 2012;54(suppl_2):S140–S5. doi: 10.1093/cid/cir1069 22403228 PMC3297553

[13] Rebuffo-Scheer C, Bose M, He J, Khaja S, Ulatowski M, Beck ET, et al. Whole genome sequencing and evolutionary analysis of human respiratory syncytial virus A and B from Milwaukee, WI 1998–2010. Plos one. 2011;6(10):e25468. doi: 10.1371/journal.pone.0025468 21998661 PMC3188560

[14] Kajon AE, Hang J, Hawksworth A, Metzgar D, Hage E, Hansen CJ, et al. Molecular epidemiology of adenovirus type 21 respiratory strains isolated from US military trainees (1996–2014). The Journal of Infectious Diseases. 2015;212(6):871–80. doi: 10.1093/infdis/jiv141 25748322

[15] Chen Y, Cui D, Zheng S, Yang S, Tong J, Yang D, et al. Simultaneous detection of influenza A, influenza B, and respiratory syncytial viruses and subtyping of influenza A H3N2 virus and H1N1 (2009) virus by multiplex real-time PCR. Journal of clinical microbiology. 2011;49(4):1653–6. doi: 10.1128/JCM.02184-10 21270233 PMC3122825

[16] Tsou T-P, Shao P-L, Lu C-Y, Chang L-Y, Kao C-L, Lee P-I, et al. Viral load and clinical features in children infected with seasonal influenza B in 2006/2007. Journal of the Formosan Medical Association. 2012;111(2):83–7. doi: 10.1016/j.jfma.2010.10.001 22370286

[17] Sedlak RH, Nguyen T, Palileo I, Jerome KR, Kuypers J. Superiority of digital reverse transcription-PCR (RT-PCR) over real-time RT-PCR for quantitation of highly divergent human rhinoviruses. Journal of clinical microbiology. 2017;55(2):442–9. doi: 10.1128/JCM.01970-16 27881615 PMC5277513

[18] Malhotra B, Swamy MA, Reddy PJ, Gupta M. Viruses causing severe acute respiratory infections (SARI) in children≤ 5 years of age at a tertiary care hospital in Rajasthan, India. The Indian Journal of Medical Research. 2016;144(6):877. 28474624 10.4103/ijmr.IJMR_22_15 PMC5433280

[19] Dare RK, Fry AM, Chittaganpitch M, Sawanpanyalert P, Olsen SJ, Erdman DD. Human coronavirus infections in rural Thailand: a comprehensive study using real-time reverse-transcription polymerase chain reaction assays. The Journal of infectious diseases. 2007;196(9):1321–8. doi: 10.1086/521308 17922396 PMC7109921

[20] Karaivanova G. Viral respiratory infections and their role as public health problem in tropical countries. African journal of medicine and medical sciences. 1995;24(1):1–7. 7495193

[21] Kwofie TB, Anane YA, Nkrumah B, Annan A, Nguah SB, Owusu M. Respiratory viruses in children hospitalized for acute lower respiratory tract infection in Ghana. Virology journal. 2012;9(1):1–8. doi: 10.1186/1743-422X-9-78 22490115 PMC3364910

[22] Lahti E, Peltola V, Waris M, Virkki R, Rantakokko-Jalava K, Jalava J, et al. Induced sputum in the diagnosis of childhood community-acquired pneumonia. Thorax. 2009;64(3):252–7. doi: 10.1136/thx.2008.099051 19052043

[23] Falsey AR, Formica MA, Walsh EE. Yield of sputum for viral detection by reverse transcriptase PCR in adults hospitalized with respiratory illness. Journal of clinical microbiology. 2012;50(1):21–4. doi: 10.1128/JCM.05841-11 22090400 PMC3256730

[24] Kelly MS, Smieja M, Luinstra K, Wirth KE, Goldfarb DM, Steenhoff AP, et al. Association of respiratory viruses with outcomes of severe childhood pneumonia in Botswana. PloS one. 2015;10(5):e0126593. doi: 10.1371/journal.pone.0126593 25973924 PMC4431806

[25] Zar HJ, Barnett W, Stadler A, Gardner-Lubbe S, Myer L, Nicol MP. Aetiology of childhood pneumonia in a well vaccinated South African birth cohort: a nested case-control study of the Drakenstein Child Health Study. The Lancet Respiratory Medicine. 2016;4(6):463–72. doi: 10.1016/S2213-2600(16)00096-5 27117547 PMC4989125

[26] Thompson WW, Shay DK, Weintraub E, Brammer L, Cox N, Anderson LJ, et al. Mortality associated with influenza and respiratory syncytial virus in the United States. Jama. 2003;289(2):179–86. doi: 10.1001/jama.289.2.179 12517228

[27] Falsey AR, Hennessey PA, Formica MA, Cox C, Walsh EE. Respiratory syncytial virus infection in elderly and high-risk adults. New England Journal of Medicine. 2005;352(17):1749–59. doi: 10.1056/NEJMoa043951 15858184

[28] Amand C, Tong S, Kieffer A, Kyaw MH. Healthcare resource use and economic burden attributable to respiratory syncytial virus in the United States: a claims database analysis. BMC health services research. 2018;18(1):1–15. 29678177 10.1186/s12913-018-3066-1 PMC5910575

[29] Belongia EA, King JP, Kieke BA, Pluta J, Al-Hilli A, Meece JK, et al., editors. Clinical features, severity, and incidence of RSV illness during 12 consecutive seasons in a community cohort of adults≥ 60 years old. Open forum infectious diseases; 2018: Oxford University Press US. 10.1093/ofid/ofy316 PMC6306566 30619907

[30] Falsey. Respiratory syncytial virus infection in adults. Clinical microbiology reviews. 2000;13(3):371–84. doi: 10.1128/CMR.13.3.371 10885982 PMC88938

[31] Vianna LA, Siqueira MM, Volpini LP, Louro ID, Resende PC. Seasonality, molecular epidemiology, and virulence of Respiratory Syncytial Virus (RSV): A perspective into the Brazilian Influenza Surveillance Program. PloS one. 2021;16(5):e0251361. doi: 10.1371/journal.pone.0251361 34003843 PMC8130917

[32] Larkin H. Investigational RSV Vaccine Given During Pregnancy Protects Newborns. JAMA. 2022;328(22):2201–. doi: 10.1001/jama.2022.20032 36511938

[33] Sattar SBA, Sharma S. Bacterial pneumonia. 2018.

[34] KADIROV KZ, MIRZAKARIMOV BK, KADIROV OZ, Abduvaliyeva CM. Problems of Studying the Pathological Anatomy of Pneumonia in Children Against the Background of Immunodeficiency. Eurasian Medical Research Periodical. 2022;10:91–3.

[35] Baggett HC, Watson NL, Deloria Knoll M, Brooks WA, Feikin DR, Hammitt LL, et al. Density of upper respiratory colonization with Streptococcus pneumoniae and its role in the diagnosis of pneumococcal pneumonia among children aged< 5 years in the PERCH study. Clinical Infectious Diseases. 2017;64(suppl_3):S317–S27. 28575365 10.1093/cid/cix100 PMC5850437

[36] Ghani ASA, Morrow BM, Hardie DR, Argent AC. An investigation into the prevalence and outcome of patients admitted to a pediatric intensive care unit with viral respiratory tract infections in Cape Town, South Africa. Pediatric Critical Care Medicine. 2012;13(5):e275–e81. doi: 10.1097/PCC.0b013e3182417848 22596071

[37] Stockman LJ, Reed C, Kallen AJ, Finelli L, Anderson LJ. Respiratory syncytial virus and Staphylococcus aureus coinfection in children hospitalized with pneumonia. The Pediatric infectious disease journal. 2010;29(11):1048–50. doi: 10.1097/INF.0b013e3181eb7315 20686440

[38] Quah J, Jiang B, Tan PC, Siau C, Tan TY. Impact of microbial Aetiology on mortality in severe community-acquired pneumonia. BMC Infectious Diseases. 2018;18(1):451. doi: 10.1186/s12879-018-3366-4 30180811 PMC6122562

[39] Hoffmann J, Machado D, Terrier O, Pouzol S, Messaoudi M, Basualdo W, et al. Viral and bacterial co-infection in severe pneumonia triggers innate immune responses and specifically enhances IP-10: a translational study. Scientific reports. 2016;6(1):1–13. 27922126 10.1038/srep38532 PMC5138590

[40] Li A, Sun Y, Liu Z, Xu X, Sun H, Sundell J. The influence of home environmental factors and life style on children’s respiratory health in Xi’an. Chinese Science Bulletin. 2014;59(17):2024–30.

[41] Naz L, Ghimire U. Assessing the prevalence trend of childhood pneumonia associated with indoor air pollution in Pakistan. Environmental Science and Pollution Research. 2020;27(35):44540–51. doi: 10.1007/s11356-020-10346-6 32770471

