# Supplementary material for: Aetiological agents of pneumonia among HIV and non-HIV infected children in Ghana: A case-control study
Source: PLoS One. 2024 Mar 22;19(3):e0299222. doi: 10.1371/journal.pone.0299222 (PMC10959341; doi:10.1371/journal.pone.0299222)
Supplement: S1 Checklist — (DOCX) [file pone.0299222.s001.docx]

STROBE Statement—checklist of items that should be included in reports of observational studies

|  | Item No. | Recommendation | Page  No. | Relevant text from manuscript |
| --- | --- | --- | --- | --- |
| **Title and abstract** | 1 | (*a*) Indicate the study’s design with a commonly used term in the title or the abstract | 1 | Aetiological agents of pneumonia among HIV and non-HIV infected children in Ghana: A case-control study. |
|  |  | (*b*) Provide in the abstract an informative and balanced summary of what was done and what was found | 2 | We conducted a case-control study that enrolled children with pneumonia as cases and non-pneumonia as controls. DNA/RNA was extracted from sputum samples and tested for viral and bacterial agents. We identified a significant proportion of viruses in cases than controls and these were mostly contributed to by Respiratory Syncytial Virus. *Staphylococcus aureus*, Klebsiella spp. and *Streptococcus pneumoniae*. |
| Introduction | | | |  |
| Background/rationale | 2 | Explain the scientific background and rationale for the investigation being reported | 3-4 | There have been numerous efforts made especially in developed countries to ascertain the scope of infectious pathogens occurring in HIV-infected patients as compared to non-HIV patients. However, information on the microbial aetiologies of pneumonia in sub-Saharan African countries is still limited. The expansion of molecular diagnostic tests in Africa has improved the identification of microbial pathogens in pneumonia irrespective of the HIV status. |
| Objectives | 3 | State specific objectives, including any prespecified hypotheses | 4 | The aim of this study was to determine the microbial agents associated with pneumonia in HIV and non-HIV populations and identify other associated risk factors. |
| Methods | | | |  |
| Study design | 4 | Present key elements of study design early in the paper | 4 | This was a prospective, observational case-control study that was conducted at the Komfo Anokye Teaching Hospital from July 2017 to May 2020. |
| Setting | 5 | Describe the setting, locations, and relevant dates, including periods of recruitment, exposure, follow-up, and data collection | 4 | This was a prospective, observational case-control study that was conducted at the Komfo Anokye Teaching Hospital from July 2017 to May 2020. The hospital is the second largest hospital in the Ghana with 1200 beds, and the main referral hospital for the Ashanti, Brong-Ahafo and most of the northern part of Ghana. Recruitment of study participants were done at the Pediatric Emergency Unit (PEU), Paediatric Intensive Care unit (PICU) and Paediatric HIV Clinic (PHC). |
| Participants | 6 | (*a*) *Cohort study*—Give the eligibility criteria, and the sources and methods of selection of participants. Describe methods of follow-up  *Case-control study*—Give the eligibility criteria, and the sources and methods of case ascertainment and control selection. Give the rationale for the choice of cases and controls  *Cross-sectional study*—Give the eligibility criteria, and the sources and methods of selection of participants | 5 | Cases were defined as children 3-12 months and presenting with clinical symptoms and signs of pneumonia including fever (>37.5°C), cough with or without difficulty in breathing as well as at least one of the following signs: high respiratory rate (>50 cpm), chest in-drawing, intercostal recession, stridor, pulmonary crackles, lethargy, oxygen saturation < 90% and radiological evidence of pneumonia if available. Controls were children with comparable age (+/- 3 months) to the recruited cases without history of chronic underlying disease, pneumonia, or respiratory ailment over the past one month prior to screening. |
|  |  | (*b*) *Cohort study*—For matched studies, give matching criteria and number of exposed and unexposed  *Case-control study*—For matched studies, give matching criteria and the number of controls per case | 6 | For every case that was recruited, a healthy control of comparable age was also selected from the neighboring community of the case. Neighbors and relatives of the cases assisted with this exercise. Controls were selected within 2 - 4 weeks of recruiting cases. |
| Variables | 7 | Clearly define all outcomes, exposures, predictors, potential confounders, and effect modifiers. Give diagnostic criteria, if applicable | 6 | Biodata collected from the study participants were socio-demographic variables (e.g., age, sex, occupation and educational level of caregivers), clinical information (e.g., weight, height/length, signs and symptoms of pneumonia, presence of complications, radiological features and outcomes of cases) and risk factors (e.g., method of cooking, number of persons in their households, accessibility to good ventilation and exposure to smoke e.g., tobacco). |
| Data sources/ measurement | 8* | For each variable of interest, give sources of data and details of methods of assessment (measurement). Describe comparability of assessment methods if there is more than one group | 6 | Data were collected from the participants, using a structured paper-based questionnaire. Sputum or induced sputum was collected from both cases and controls. Sputum induction was performed according to guidelines and procedures described by Hammit et al and Grant et al [11, 12]  Standard microbiological techniques were performed on sputum and blood culture samples to isolate and identify bacteria from the clinical specimens collected. |
| Bias | 9 | Describe any efforts to address potential sources of bias | 5 | Controls were randomly drawn from the same catchment area of the cases and invited to the healthcare facility for sample collection. |
| Study size | 10 | Explain how the study size was arrived at | 11 | We estimated a samples size of 179 individuals each for cases and controls. This estimate was based on previous RSV detections of 21.3% being the most predominant virus among children (Kwofie et al., 2018) and estimated 10% exposure among controls (Kwofie et al., 2018). The estimated sample size determined was based on a power of 80%, a two-sided alpha error of 5%. |

Continued on next page

| Quantitative variables | 11 | Explain how quantitative variables were handled in the analyses. If applicable, describe which groupings were chosen and why | 11 | Descriptive statistics were computed for continuous and categorical variables.  Results were expressed as odds ratio with 95% confidence interval. |
| --- | --- | --- | --- | --- |
| Statistical methods | 12 | (*a*) Describe all statistical methods, including those used to control for confounding | 11 | Data were entered into Microsoft Excel and exported to R statistical software (version 3.6.1) for analysis. Statistical comparisons between subgroups of categorical variables were analyzed using the Fischer's exact test or Chi-square test where appropriate. A multivariate logistic regression was used to determine the association between microorganisms detected and pneumonia risk. Multivariate models were adjusted for microorganisms detected in induced sputum separately for HIV and non-HIV patients. |
|  |  | (*b*) Describe any methods used to examine subgroups and interactions | 11 | Statistical comparisons between subgroups of categorical variables were analyzed using the Fischer's exact test or Chi-square test where appropriate. |
|  |  | (*c*) Explain how missing data were addressed |  | None reported |
|  |  | (*d*) *Cohort study*—If applicable, explain how loss to follow-up was addressed  *Case-control study*—If applicable, explain how matching of cases and controls was addressed  *Cross-sectional study*—If applicable, describe analytical methods taking account of sampling strategy | 6 | For every case that was recruited, a healthy control of comparable age was also selected from the neighboring community of the case.  Controls were selected within 2 - 4 weeks of recruiting cases. |
|  |  | (*e*) Describe any sensitivity analyses |  | Not applicable |
| Results | | | | |
| Participants | 13* | (a) Report numbers of individuals at each stage of study—eg numbers potentially eligible, examined for eligibility, confirmed eligible, included in the study, completing follow-up, and analysed | 11, 13 | The study recruited 404 individuals with males being 232 (58.3%). Of the 404 recruited, 148 (36.6%) were HIV infected persons and among them 57.4% (85/148) presented with pneumonia. Details shown in Table 2 |
|  |  | (b) Give reasons for non-participation at each stage |  | Not applicable |
|  |  | (c) Consider use of a flow diagram |  | Not available |
| Descriptive data | 14* | (a) Give characteristics of study participants (eg demographic, clinical, social) and information on exposures and potential confounders | 13 | Shown in Table 3 |
|  |  | (b) Indicate number of participants with missing data for each variable of interest |  | Not applicable |
|  |  | (c) *Cohort study*—Summarise follow-up time (eg, average and total amount) |  | Not applicable |
| Outcome data | 15* | *Cohort study*—Report numbers of outcome events or summary measures over time |  | Not applicable |
|  |  | *Case-control study—*Report numbers in each exposure category, or summary measures of exposure | 13, 15, 17 | Shown in Tables 2, 3, 4 |
|  |  | *Cross-sectional study—*Report numbers of outcome events or summary measures |  | Not applicable |
| Main results | 16 | (*a*) Give unadjusted estimates and, if applicable, confounder-adjusted estimates and their precision (eg, 95% confidence interval). Make clear which confounders were adjusted for and why they were included | 11 | Shown in Tables 3, 4, 5, 6 |
|  |  | (*b*) Report category boundaries when continuous variables were categorized | 10, 11 | Shown in Tables 2 and 3 |
|  |  | (*c*) If relevant, consider translating estimates of relative risk into absolute risk for a meaningful time period |  | Not applicable |

Continued on next page

| Other analyses | 17 | Report other analyses done—eg analyses of subgroups and interactions, and sensitivity analyses | 14, 16 | Association between clinical presentations and microbial detections have been shown in Supplementary Tables 1-3. |
| --- | --- | --- | --- | --- |
| Discussion | | | | |
| Key results | 18 | Summarise key results with reference to study objectives | 20, 21, 22 | RSV was observed as the main virus associated with pneumonia in both HIV and non-HIV patients.  Assessment of sputum or induced sputum specimens using conventional cultures revealed a high number of bacterial organisms in pneumonia cases compared to controls. The most predominant microbial organism isolated was Klebsiella spp., followed by Streptococcus pneumoniae and Staphylococcus aureus. For both HIV and non-HIV populations, Klebsiella spp. was found to be significantly predominant in cases compared to controls.  In terms of socio-demographic characteristics of our study populations, we observed HIV infected children exposed to cigarette and non-HIV children involved in indoor cooking and other modes were more associated with pneumonia. |
| Limitations | 19 | Discuss limitations of the study, taking into account sources of potential bias or imprecision. Discuss both direction and magnitude of any potential bias | 22 | One major limitation of the study is our inability to use molecular techniques to investigate non-viral microbial organisms. The use of these advanced methods could have allowed for proper estimation of the full spectrum of other non-viral agents involved in the causal pathway of pneumonia  The difficulty in enrolling healthy people to provide induced sputum limited our ability to recruit large number of persons for this study. An imbalance in the case-control ratio numbers could have impact on the results. |
| Interpretation | 20 | Give a cautious overall interpretation of results considering objectives, limitations, multiplicity of analyses, results from similar studies, and other relevant evidence | 20-22 | “Discussion” section in the manuscript |
| Generalisability | 21 | Discuss the generalisability (external validity) of the study results | 23 | This study has emphasized the importance of microbial organisms particularly RSV in contributing to pneumonia among children with/without HIV. Identification of these pathogens has improved our knowledge on the burden of respiratory pathogens in Africa. The study has provided further evidence about the need for further advocacy for vaccines to improve the wellbeing of children in Africa. |
| Other information | |  | | |
| Funding | 22 | Give the source of funding and the role of the funders for the present study and, if applicable, for the original study on which the present article is based | 23 | This project is part of the EDCTP2 programme supported by the European Union (grant number TMA 2015 CDF - 1033) and awarded under the Career Development Fellowship to Dr Michael Owusu. |

*Give information separately for cases and controls in case-control studies and, if applicable, for exposed and unexposed groups in cohort and cross-sectional studies.

**Note:** An Explanation and Elaboration article discusses each checklist item and gives methodological background and published examples of transparent reporting. The STROBE checklist is best used in conjunction with this article (freely available on the Web sites of PLoS Medicine at http://www.plosmedicine.org/, Annals of Internal Medicine at http://www.annals.org/, and Epidemiology at http://www.epidem.com/). Information on the STROBE Initiative is available at www.strobe-statement.org.
