# Supplementary material for: Aetiological agents of pneumonia among HIV and non-HIV infected children in Ghana: A case-control study
Source: PLoS One. 2024 Mar 22;19(3):e0299222. doi: 10.1371/journal.pone.0299222 (PMC10959341; doi:10.1371/journal.pone.0299222)
Supplement: S1 Table — (PDF) [file pone.0299222.s002.pdf]

S1 Table. Clinical Presentations Associated with Microbial Detection for all cases

Clinical Presentation	Viruses detected (%)			Bacteria detected (%)			Viral-Bacterial Co-infection (%)		
	**Negative**	**Positive**	**P value**	**Negative**	**Positive**	**P value**	**Negative**	**Positive**	**P value**
Total	105	125		97	18		87	28	
Shortness of breath	6 (8.8)	7 (9)	1	5 (8.1)	0 (0)	1	3 (5.2)	2 (15.4)	0.224
Poor feeding	36 (36)	37 (31.4)	0.562	30 (33.7)	6 (37.5)	0.993	23 (29.1)	13 (50)	0.088
Diarrhoea	10 (10.2)	14 (12.1)	0.831	9 (10.3)	1 (6.2)	1	5 (6.4)	5 (20)	0.06
Vomiting	25 (25.3)	25 (21.2)	0.585	19 (21.3)	7 (43.8)	0.067	21 (26.2)	5 (20)	0.714
Rhinorrhoea	34 (34.3)	39 (33.3)	0.99	24 (27)	3 (18.8)	0.757	19 (24.1)	8 (30.8)	0.674
Fast breathing	40 (41.2)	57 (48.7)	0.339	32 (36.4)	5 (33.3)	1	55 (69.6)	11 (45.8)	0.06
Chest recession	34 (35.8)	39 (34.2)	0.926	21 (24.4)	5 (33.3)	0.526	17 (22.1)	9 (37.5)	0.214
Lethargy	18 (18.2)	16 (14)	0.524	10 (11.5)	3 (18.8)	0.421	9 (11.2)	4 (17.4)	0.48
Pulmonary crackles	36 (36.4)	43 (37.1)	1	27 (30.7)	5 (31.2)	1	25 (31.2)	7 (29.2)	1
Wheeze	85 (86.7)	90 (78.3)	0.153	75 (85.2)	14 (87.5)	1	69 (86.2)	20 (83.3)	0.744
Flaring of ala nasi	43 (44.8)	61 (52.6)	0.321	31 (34.8)	8 (50)	0.382	25 (31.2)	14 (56)	**0.046**
Lower chest indrawing	42 (43.8)	51 (45.1)	0.952	26 (30.2)	7 (43.8)	0.441	21 (26.6)	12 (52.2)	**0.04**
Oxygen required for 48hours	13 (20)	14 (15.7)	0.636	11 (19.6)	3 (30)	0.431	10 (20.8)	4 (22.2)	1
Severe pneumonia	94 (92.2)	114 (96.6)	0.249	85 (93.4)	15 (93.8)	1	74 (91.4)	26 (100)	0.191
Very severe pneumonia	43 (47.8)	53 (52)	0.664	28 (35.9)	7 (43.8)	0.758	22 (30.1)	13 (61.9)	**0.017**
