# Supplementary material for: Aetiological agents of pneumonia among HIV and non-HIV infected children in Ghana: A case-control study
Source: PLoS One. 2024 Mar 22;19(3):e0299222. doi: 10.1371/journal.pone.0299222 (PMC10959341; doi:10.1371/journal.pone.0299222)
Supplement: S2 Table — (PDF) [file pone.0299222.s003.pdf]

S2 Table. Clinical Presentation Associated with Microbial Detection in HIV Patients with Pneumonia

**Clinical Presentation**	**Viruses detected** (%)			**Bacteria detected (%)**			**Viral-Bacterial Co-infection (%)**		
	**Negative**	**Positive**	**P value**	**Negative**	**Positive**	**P value**	**Negative**	**Positive**	**P value**
Total	86	61		91	9		84	16	
Shortness of breath	2 (5.6)	1 (2.9)	1	2 (5)	0 (0)	1	1 (2.6)	1 (14.3)	0.284
Poor feeding	4 (10.3)	7 (17.9)	0.515	7 (14.9)	0 (0)	0.582	2 (4.8)	5 (45.5)	**0.003**
Diarrhoea	4 (10.5)	4 (10.3)	1	4 (8.7)	1 (16.7)	0.473	3 (7.3)	2 (18.2)	0.283
Vomiting	3 (7.7)	2 (5.1)	1	4 (8.5)	1 (16.7)	0.465	4 (9.5)	1 (9.1)	1
Rhinorrhoea	4 (10.3)	5 (12.8)	1	4 (8.5)	0 (0)	1	2 (4.8)	2 (18.2)	0.187
Fast breathing	3 (7.7)	8 (21.1)	0.177	6 (13)	1 (16.7)	1	2 (4.8)	5 (50)	**0.002**
Chest recession	5 (13.9)	6 (16.7)	1	7 (15.9)	0 (0)	0.576	3 (7.5)	4 (40)	**0.023**
Lethargy	3 (7.7)	3 (8.6)	1	4 (8.9)	0 (0)	1	3 (7.1)	1 (11.1)	0.552
Pulmonary crackles	7 (17.9)	16 (44.4)	**0.025**	14 (31.1)	0 (0)	0.17	12 (28.6)	2 (22.2)	1
Wheeze	0 (0)	2 (5.6)	0.233	2 (4.3)	0 (0)	1	2 (4.8)	0 (0)	1
Flaring of ala nasi	5 (13.5)	6 (16.7)	0.961	7 (15.2)	1 (16.7)	1	5 (11.9)	3 (30)	0.171
Lower chest indrawing	4 (10.8)	4 (11.8)	1	5 (11.1)	0 (0)	1	2 (4.8)	3 (33.3)	**0.033**
Oxygen required for 48hours	1 (9.1)	2 (12.5)	1				2 (15.4)	1 (14.3)	1
Severe pneumonia	38 (92.7)	39 (100)	0.241	47 (95.9)	5 (83.3)	0.298	41 (93.2)	11 (100)	1
Very severe pneumonia	4 (11.8)	4 (12.1)	1	5 (11.9)	0 (0)	1	2 (5)	3 (37.5)	**0.027**
