# Supplementary material for: Aetiological agents of pneumonia among HIV and non-HIV infected children in Ghana: A case-control study
Source: PLoS One. 2024 Mar 22;19(3):e0299222. doi: 10.1371/journal.pone.0299222 (PMC10959341; doi:10.1371/journal.pone.0299222)
Supplement: S3 Table — (PDF) [file pone.0299222.s004.pdf]

S3 Table. Clinical Presentation Associated with Microbial Detection in Non-HIV Patients

Clinical Presentation	Viruses detected (%)			Bacteria detected (%)			Virus-Bacteria Co-infection (%)		
	**Negative**	**Positive**	**P value**	**Negative**	**Positive**	**P value**	**Negative**	**Positive**	**P value**
Total	106	125		68	15		64	19	
Shortness of breath	4 (12.5)	6 (13.6)	1	3 (13.6)	0 (0)	1	2 (10.5)	1 (16.7)	1
Poor feeding	32 (52.5)	30 (38)	0.124	23 (54.8)	6 (60)	1	21 (56.8)	8 (53.3)	1
Diarrhoea	6 (10)	10 (13)	0.786	5 (12.2)	0 (0)	0.569	2 (5.4)	3 (21.4)	0.12
Vomiting	22 (36.7)	23 (29.1)	0.447	15 (35.7)	6 (60)	0.282	17 (44.7)	4 (28.6)	0.462
Rhinorrhoea	30 (50)	34 (43.6)	0.564	20 (47.6)	3 (30)	0.482	17 (45.9)	6 (40)	0.934
Fast breathing	37 (63.8)	49 (62)	0.974	26 (61.9)	4 (44.4)	0.46	22 (59.5)	8 (57.1)	1
Chest recession	29 (49.2)	33 (42.3)	0.533	14 (33.3)	5 (55.6)	0.266	14 (37.8)	5 (35.7)	1
Lethargy	15 (25)	13 (16.5)	0.303	6 (14.3)	3 (30)	0.349	6 (15.8)	3 (21.4)	0.688
Pulmonary crackles	29 (48.3)	27 (33.8)	0.117	13 (30.2)	5 (50)	0.279	13 (34.2)	5 (33.3)	1
Wheeze	13 (21.7)	23 (29.1)	0.425	11 (26.2)	2 (20)	1	9 (23.7)	4 (28.6)	0.729
Flaring of ala nasi	38 (64.4)	55 (68.8)	0.722	24 (55.8)	7 (70)	0.494	20 (52.6)	11 (73.3)	0.285
Lower chest indrawing	38 (64.4)	47 (59.5)	0.682	21 (51.2)	7 (70)	0.48	19 (51.4)	9 (64.3)	0.608
Oxygen required for 48hours	12 (22.2)	12 (16.4)	0.553	8 (22.2)	3 (30)	0.682	8 (22.9)	3 (27.3)	1
Severe pneumonia	56 (91.8)	75 (94.9)	0.503	38 (90.5)	10 (100)	0.576	33 (89.2)	15 (100)	0.311
Very severe pneumonia	39 (69.6)	49 (71)	1	23 (63.9)	7 (70)	1	20 (60.6)	10 (76.9)	0.493
